# The Role of Element Segregation in the Fracture Mechanism and Performance of Spot-Welded AlSi_7_MnMg Aluminum Alloy Joints

**DOI:** 10.3390/ma19040747

**Published:** 2026-02-14

**Authors:** Hong Xu, Miao Zhao, Rui Wang, Lijun Han, Xiuming Cheng, You Fang

**Affiliations:** 1 Key Laboratory of Automotive Materials Ministry of Education, School of Material Science and Technology, Jilin University, Changchun 130022, China; xh@jlu.edu.cn; 2 College of Materials Science and Engineering, Jilin University, Changchun 130025, China; miaoz23@mails.jlu.edu.cn (M.Z.); ruiw22@mails.jlu.edu.cn (R.W.); xmcheng@jlu.edu.cn (X.C.); youf24@mails.jlu.edu.cn (Y.F.); 3 FAW-Volkswagen Automotive Co., Ltd., Changchun 130011, China

**Keywords:** resistance spot welding, aluminium alloy, fracture mechanism, element segregation

## Abstract

This study systematically investigates the microstructural characteristics and mechanical properties of resistance spot-welded joints in 3 mm thick non-heat-treatable die-cast AlSi_7_MnMg alloy, with particular focus on the influence of element segregation and secondary phase behavior on fracture mechanisms and the process window. The results indicate that the weld nugget exhibits a typical dual structure consisting of columnar and equiaxed grain zones, with a corresponding “M”-shaped microhardness profile. Significant segregation of Si, Fe, and Mn elements at the nugget boundary was observed, leading to the formation of low-melting-point eutectic regions and secondary phase bands. These features induce microporosity along segregation trajectories, serving as crack initiation sites and resulting in a notably narrowed spot welding process window. From the perspective of microstructure and solute behavior during non-equilibrium solidification, this work elucidates the intrinsic mechanisms governing joint performance and process stability in non-heat-treatable die-cast aluminum alloys, providing a theoretical basis for their engineering applications.

## 1. Introduction

Driven by the global imperative for energy savings, emission reduction, and sustainable development, the automotive industry is undergoing a technological transformation centered on lightweighting and integrated manufacturing. Within this context, non-heat-treatable die-cast aluminum alloys have emerged as ideal materials for manufacturing large, thin-walled, and complex structural components. The primary advantage of these alloys (e.g., AlSi_7_MnMg) lies in their good mechanical properties in the as-cast state, eliminating the need for subsequent solution and aging treatments. This fundamentally avoids defects such as warping, distortion, and surface blistering associated with heat treatment processes, thereby significantly enhancing production efficiency and dimensional stability. However, following integrated die-casting, these large components still require reliable joining techniques to be assembled with other body-in-white parts. Resistance spot welding remains an indispensable joining technology in automotive body manufacturing due to its high efficiency, low cost, and ease of automation. Consequently, investigating the feasibility, joint quality, and process stability of resistance spot welding for non-heat-treatable die-cast aluminum alloys is of critical importance for their successful industrial implementation [1,2].

Compared to conventional wrought aluminum alloys, alloys like AlSi_7_MnMg typically contain higher levels of silicon and manganese to fulfill die-castability and non-heat-treatable requirements. This results in an inherent as-cast microstructure featuring coarse eutectic silicon phases and complex Fe/Mn-rich intermetallic compounds. These microstructural characteristics impart distinct physico-metallurgical properties: Firstly, the presence of secondary phase particles significantly reduces electrical conductivity, affecting heat generation and distribution efficiency during spot welding. Secondly, the wide solidification temperature range coupled with a pronounced tendency for elemental segregation collectively leads to high hot cracking susceptibility. These inherent material properties promote the formation of defects such as porosity, shrinkage voids, and solidification cracks along grain boundaries in the weld nugget during the rapid melting and solidification cycle of spot welding. Ultimately, this manifests as a significantly narrower processing window for achieving high-quality spot welds compared to traditional wrought aluminum alloys, severely constraining joint mechanical performance and process stability in mass production.

Extensive research has been conducted on the resistance spot welding of conventional aluminum alloys, particularly those used in automotive applications, leading to substantial progress in process optimization, microstructure-property relationships, and failure mechanisms [3,4,5]. In contrast, research on the spot welding of non-heat-treatable die-cast aluminum alloys remains in its infancy. The current knowledge gaps are primarily threefold: First, the complex interaction between the unique as-cast initial microstructure of the base material and the nugget microstructure formed under rapid non-equilibrium solidification during spot welding, and its precise influence on solidification behavior and final microstructural characteristics, is not yet fully understood. Second, the specific role of these microstructural features—particularly the formation of elemental segregation bands and brittle intermetallic phases at the nugget boundary—in governing the macroscopic mechanical properties and lap-shear failure behavior of the joints requires further elucidation. Third, a fundamental theoretical basis for targeted process optimization to widen the welding window and suppress defects is still lacking.

To address these gaps, this study systematically investigates the resistance spot welds of a 3 mm thick AlSi_7_MnMg non-heat-treatable die-cast aluminum alloy, a material with significant application potential in the automotive industry. Through metallographic statistics, SEM-EDS surface scanning, and phase identification, we established a correlation map between as-cast microstructure characteristics and melt core microstructure types by comparing phase compositions, size distributions, and grain morphologies across different regions of the base material and melt core. For segregation zones, we conducted component gradient measurements, identified second-phase categories, analyzed porosity defect distributions, and performed spatial correlation analysis with corresponding microhardness distributions and tensile/shear fracture morphology. We established causal relationships between element segregation zones and joint failure modes, clarifying the crack initiation pathways through segregation. A process window diagram (current, pressure, time) was developed with dual constraints of “minimum allowable melt core diameter” and “optimal melt core diameter,” along with optimized process parameter criteria based on segregation suppression efficiency. This provides actionable operational guidance for practical production. The research aims to establish reliable theoretical foundations and practical implementation guidelines for resistance spot welding processes and quality control of heat-treated die-cast aluminum alloys, thereby promoting the reliable application of this advanced material and manufacturing technology in high-end equipment manufacturing.

## 2. Experiments and Methods

**Figure 1:**
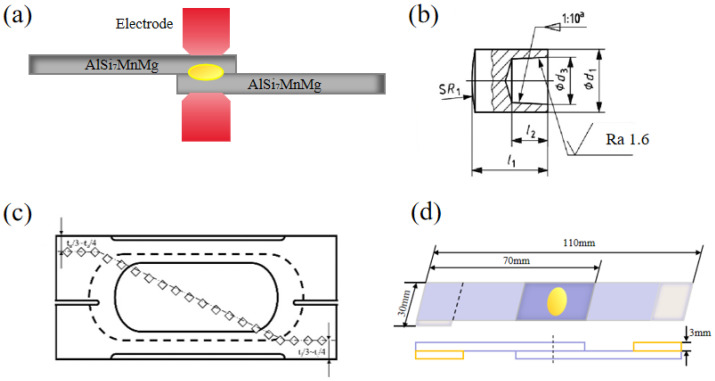
(**a**) Schematic of the resistance spot welding process; (**b**) Electrode cap dimensions; (**c**) Schematic of sampling locations; (**d**) Shear test dimensions.

**Figure 2:**
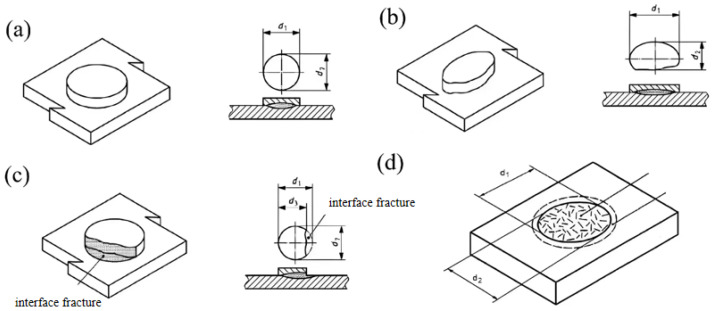
Schematic diagrams of welding point diameter d_w_ measurement: (**a**) Symmetric separation fracture; (**b**) Asymmetric separation fracture; (**c**) Mixed fracture; (**d**) Shear fracture.

The test material used in this study is a 3 mm thick AlSi_7_MnMg vacuum die-cast non-heat-treated aluminum alloy plate, with its chemical composition shown in Table 1 below. As illustrated in Figure 1a, resistance spot welding was employed for the non-heat-treated die-cast aluminum alloy. The welding workstation utilized a KUKAR270 welding robot paired with an HWH MF 1000 welding controller manufactured by NIMAK, equipped with X-type NIMAK C8000 welding clamps produced by HARMS + WENDE, rated at 2 × 178 KVA with a 20% duty cycle. The test adopted a flat plate lap configuration. Prior to welding, the plates were cut to specified dimensions (40 mm × 140 mm × 3 mm for fracture inspection and metallographic analysis, 30 mm × 70 mm × 3 mm for tensile shear testing) according to corporate standards. Subsequently, mechanical grinding, acetone degreasing, and ethanol ultrasonic cleaning were performed to thoroughly remove surface oxidation layers and oil contaminants, ensuring contact surface quality. The resistance spot welding electrodes, made of widely used chromium-zirconium copper material (as shown in Figure 1b), strictly adhered to ISO 05821-2009 specifications, with a diameter of 20 mm and a fillet radius of 100 mm. To ensure welding quality, based on preliminary test results, the welding point edge spacing was set at 20 mm and the welding point spacing at 50 mm to effectively avoid edge effects and current diversion phenomena. During welding, the cooling water flow rates for the welding gun circuit and inverter were set at 8 L/min and 4 L/min, respectively. To maintain consistent welding quality, electrode caps were polished after each welding point was completed. The welding point diameter was calculated as d_w_ = (d_1_ + d_2_)/2, where d_1_ is the maximum diameter of the welding point and d_2_ is the minimum diameter. As shown in Figure 2, measurement methods were determined based on welding point failure modes: (1) For shear fracture, d_w_ represents the average diameter of the fracture surface at the workpiece bonding plane (excluding adhesive regions); (2) For detachment fracture, d_w_ is the average diameter of the bottom surface of the detached boss; (3) For mixed fracture, d_w_ is the average diameter of the bottom surface of the detached boss (including fracture portions within the bonding plane); (4) For special cases where failure occurs at the base material position, the fusion core diameter must be measured through metallographic testing.

Based on the aforementioned research, welding experiments were designed to determine the nugget diameter under different welding currents and electrode forces at a fixed welding time of 100 ms. The weldability window test shall be performed in accordance with the current international standard ISO 14327:2004.

## 3. Results and Discussion

### 3.1. Morphological and Mechanical Properties

**Figure 3:**
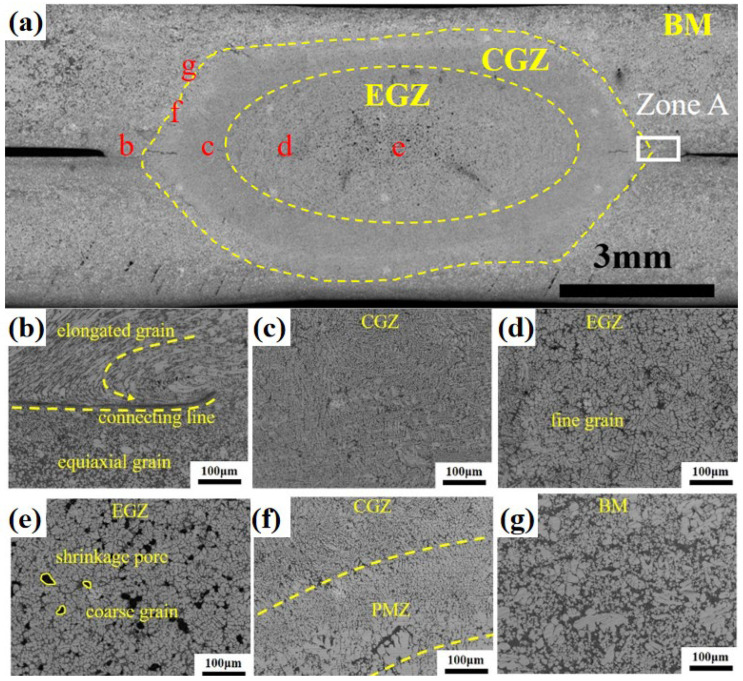
(**a**) Microstructural characteristics of the resistance spot welding joint of heat-treatment-free die-casting aluminum alloy; (**b**) Connecting line; (**c**) Columnar grain zone; (**d**) Equiaxial fine grain zone; (**e**) Equiaxial coarse grain zone; (**f**) Partially melted zone; (**g**) Base Metal.

Metallographic specimens were fabricated for the weld point under the conditions of I = 40 kA, F = 6.5 kN, and t = 100 ms. Cross-sectional morphology was observed using a stereomicroscope, revealing the microstructure characteristics of the heat-treatment-free die-cast aluminum alloy resistance spot weld joint, as shown in Figure 3a. The fusion core exhibited a disc-shaped morphology with a distinct shrinkage cavity at its center, a phenomenon caused by the inherent high solidification shrinkage rate of aluminum alloys and an inevitable aspect of the resistance spot welding process. Macroscopic examination revealed no common spot welding defects such as interface spatter, porosity, or hot cracks, confirming the success of this resistance spot welding experiment. Measured values for this weld include a nugget diameter of 9.87 mm, a nugget height of 4.84 mm, and an indentation depth of 0.22 mm. All these parameters exceed the stipulated standards, indicating excellent performance in both visual quality and structural integrity. The weld nugget primarily consists of a central equiaxed grain zone (EGZ) and a peripheral columnar grain zone (CGZ). The EGZ corresponds to the central region containing the shrinkage cavity. A contrast variation is visible within the CGZ, resulting from the decreased thermal influence experienced by its outer regions, which are located farther from the nugget center during the welding process.

To thoroughly examine the joint microstructure, magnified views of the regions in Figure 3a are provided. Figure 3b shows the bond line area of the resistance spot weld, which entered a thermoplastic state under thermal cycles, deforming and bonding under electrode pressure. The bond line exhibits slight curvature at its end, with notably different grain morphology on both sides. Compared to the base metal microstructure in Figure 3g, grains in the upper sheet are significantly elongated, forming a streamlined structure, while those in the lower sheet are refined into equiaxed grains. This difference arises mainly from uneven pressure distribution along the thickness direction: the upper electrode applies predominant pressure, and enhanced fluidity at high temperatures promotes grain flow along specific directions. In contrast, the lower electrode primarily provides support with less pressure. Under rapid thermo-mechanical coupling, the lower sheet undergoes severe thermoplastic deformation, accumulating high dislocation density and distortion energy, which drives recrystallization. Subsequently, at peak temperature, nucleation occurs mainly via grain boundary bulging or subgrain coalescence. The new nuclei grow rapidly into equiaxed grains at high temperatures, ultimately replacing the deformed microstructure with strain-free equiaxed recrystallized grains [6,7,8].

Figure 3c displays the outer CGZ of the nugget, dominated by typical columnar grains. Figure 3d,e show the central EGZ, revealing a mixed “columnar + equiaxed” structure. According to solidification theory [9,10,11], the high cooling rate near the water-cooled electrodes at the nugget boundary and the presence of partially melted grains provided favorable conditions for the heterogeneous nucleation of columnar grains. These grains grew epitaxially towards the nugget center, aligning with the maximum temperature gradient. During growth, solute elements (e.g., Si) were rejected into the interdendritic regions and the solid–liquid interface front, leading to local enrichment and increased constitutional undercooling, which further promoted the extension of columnar grains. As fragmented dendrites were transported into the nugget center by fluid flow, they served as nucleation sites for equiaxed dendritic grains in the region characterized by a lower temperature gradient and slower cooling rate. This process ultimately resulted in the formation of the composite “columnar + equiaxed” structure. Although both Figure 3d,e belong to the EGZ, the region in (d) exhibits finer grains and fewer shrinkage pores. This difference stems from the spatial heterogeneity of the thermal cycle during spot welding. Variations in cooling rate influenced the nucleation/growth mechanisms and feeding behavior, consequently leading to the distinct grain size and defect density observed between the two regions. Figure 3f illustrates the microstructure at the edge of the nugget’s CGZ, showing the partially melted zone and the transition from cellular to columnar grains within it.

**Figure 4:**
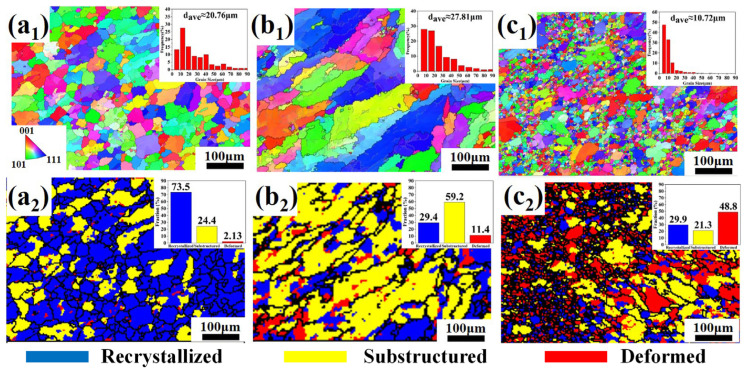
IPF maps and recrystallization distribution at different locations of the resistance spot welding joint: (**a_1_**) IPF map of the equiaxed grain zone; (**b_1_**) IPF map of the columnar grain zone; (**c_1_**) IPF map of the fusion boundary zone; (**a_2_**) Recrystallization distribution map of the equiaxed grain zone; (**b_2_**) Recrystallization distribution map of the columnar grain zone; (**c_2_**) Recrystallization distribution map of the fusion boundary zone.

Figure 4 presents the EBSD analysis results of different regions within the spot weld fusion core. Significant differences in grain size and morphology were observed across these zones: the outer columnar grains were markedly larger than the central equiaxed zone, while the edge region contained numerous fine, dense, and uniformly oriented equiaxed grains. Statistical analysis of Figure 4a_1_–c_1_ shows that the average grain sizes in the central equiaxed zone and columnar zone were 20.8 μm and 27.8 μm, respectively, whereas the edge fine equiaxed zone averaged 10.7 μm. Compared to the base material (average 13.4 μm), the post-weld fusion core exhibited grain coarsening internally and significant grain refinement at the edges, primarily due to substantial plastic deformation during welding.

Under thermomechanical coupling, the recrystallization characteristics in different regions of the melt core undergo significant changes. As shown in Figure 4a_2_, the melt core center predominantly exhibits recrystallized grains, with its recrystallization fraction increasing by 40.6% compared to the parent material, while the subgrain fraction decreases by 36.3%. This is attributed to the region experiencing high temperature, high strain rate, and rapid cooling, which promotes dynamic recrystallization. The columnar grain region, on the other hand, is dominated by subgrains (59.2%), with the proportion of deformed grains increasing by 9.3% compared to the melt core center (Figure 4b_2_). This occurs because the columnar grain region is closer to the melt core boundary, where better heat dissipation conditions prevent sufficient recrystallization and recovery under faster cooling rates. Additionally, constrained by the adjacent parent material, free shrinkage is limited, further intensifying plastic deformation and increasing the proportion of deformed grains. The fine equiaxed grain region at the melt core edge is primarily composed of deformed grains (53.4%), with its equiaxed grain fraction approaching that of the columnar grain region (Figure 4c_2_). This region has not undergone complete melting, is significantly affected by electrode mechanical actions, and experiences further grain deformation due to thermal stress during cooling.

**Figure 5:**
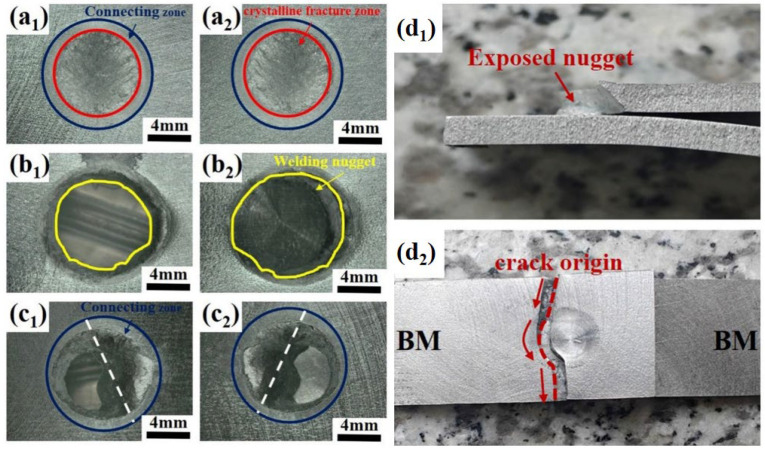
Failure modes of Al_7_SiMnMg resistance spot welding joints: (**a_1_**,**a_2_**) Interfacial fracture; (**b_1_**,**b_2_**) Pull-out fracture; (**c_1_**,**c_2_**) Mixed fracture; (**d_1_**,**d_2_**) The failure located in the base material.

Figure 5 illustrates the typical failure modes of spot-welded joints in the AlSi_7_MnMg non-heat-treatable die-cast aluminum alloy under lap-shear testing, which primarily include interfacial fracture (IF), pull-out fracture (PF), and a mixed mode exhibiting characteristics of both. Figure 5a_1_,a_2_ show the interfacial fracture morphology obtained under the welding parameters of I = 32.5 kA, F = 7.5 kN, and t = 100 ms. The maximum lap-shear load for this joint was 5124.63 N, which failed to meet the minimum lap-shear strength requirement specified for 3 mm thick sheets. Interfacial fracture typically occurs in low-strength spot welds, and the fracture surface consists of an outer bonded interface region and an inner crystalline fracture surface. Figure 5b_1_,b_2_ correspond to the pull-out fracture morphology obtained under the parameters I = 37.5 kA, F = 6.5 kN, and t = 75 ms. This mode is characterized by the complete extraction of the intact weld nugget from one of the sheets and is commonly observed in high-strength spot welds. The corresponding maximum lap-shear load was 6588.43 N, satisfying and exceeding the minimum strength requirement for 3 mm sheets. Figure 5c_1_,c_2_ present the mixed fracture morphology obtained with I = 37.5 kA, F = 6.0 kN, and t = 100 ms, which exhibited a maximum lap-shear load of 7640.27 N.

In some instances, fracture occurred in the base metal during lap-shear testing, and the associated load typically exceeded that of conventional pull-out fractures. As shown in Figure 5d_1_,d_2_, under the parameters I = 40 kA, F = 6.5 kN, and t = 100 ms, the joint achieved a maximum lap-shear load of 8518.89 N, significantly surpassing the minimum requirement for 3 mm sheets and reflecting excellent joint strength and reliability. Base metal failure typically initiates at the sheet edge, away from the weld nugget, where stress concentration is more prone to occur under tensile loading. With increasing stress, a crack initiates and propagates. The figures indicate that the crack extends perpendicularly from the sheet edge and, upon encountering the weld nugget, deflects and propagates along the nugget periphery. The final fracture surface reveals a portion of the nugget microstructure. Base metal failure signifies that the strength of the weld nugget approaches that of the base material, representing an ideal failure mode [12].

**Figure 6:**
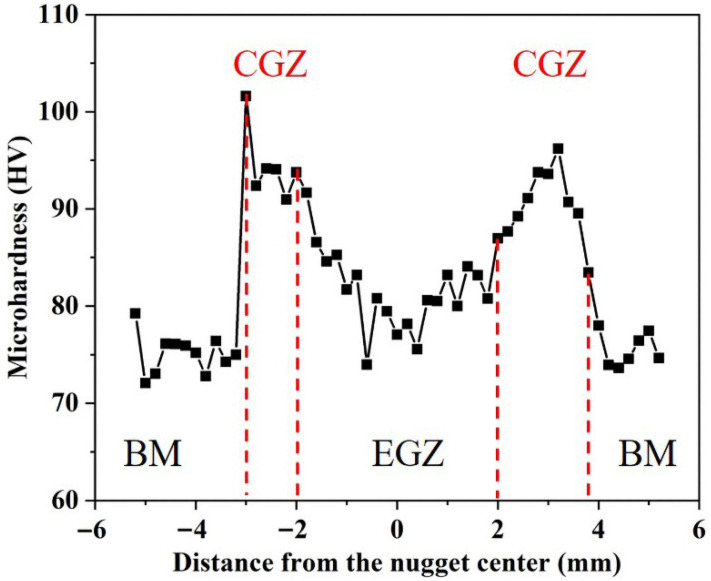
Typical microhardness profile of the AlSi_7_MnMg spot-welded joint.

Figure 6 shows the Vickers microhardness distribution across the resistance spot weld of the non-heat-tractable die-cast aluminum alloy. The profile exhibits a distinct “M” shape along the indentation path, which traverses BM, CGZ, EGZ, another CGZ, and the opposing BM. The average microhardness of the BM is approximately 75.3 HV. The peak hardness of 101.6 HV is observed within the CGZs, with an average value of about 91.8 HV in these zones, representing an increase of 21.9% compared to the BM. In contrast, the EGZ shows significant softening, with the microhardness dropping to an average of approximately 81.0 HV.

### 3.2. Study of the Process Window for Spot-Welded Joints

**Figure 7:**
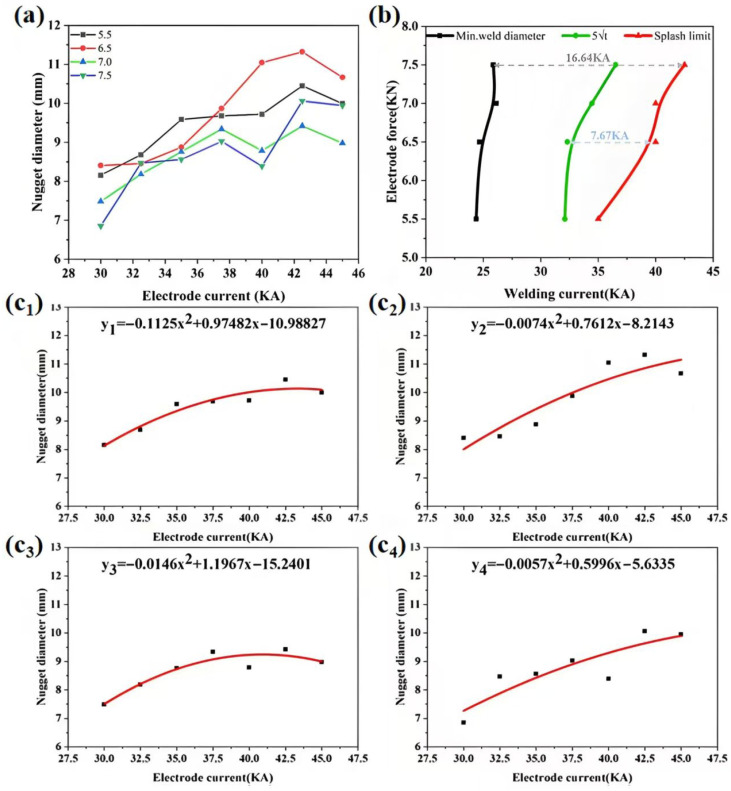
(**a**) Experimental data from the weldability tests; (**b**) Weldability lobe depicting the operating window of welding current versus electrode force; (**c_1_**–**c_4_**) Quadratic fits of the relationship between welding current and nugget diameter under different electrode forces: (**c_1_**) 5.5 kN, (**c_2_**) 6.5 kN, (**c_3_**) 7.0 kN, (**c_4_**) 7.5 kN.

Based on the aforementioned research, welding experiments were designed to determine the nugget diameter under different welding currents and electrode forces at a fixed welding time of 100 ms. The results are shown in Figure 7a. The minimum welding currents required to initiate interfacial expulsion under different electrode forces were also recorded. The results indicate that the minimum expulsion current increases with higher electrode force, specifically: the minimum expulsion currents corresponding to electrode forces of 5.5 kN, 6.5 kN, 7.0 kN, and 7.5 kN are 35 kA, 40 kA, 40 kA, and 42.5 kA, respectively.

The obtained data were fitted with quadratic curves, and the fitting results are shown in Figure 7c_1_–c_4_. The fitting functions for the different electrode forces are given below, where *y* represents the nugget diameter and *x* denotes the welding current:

(1) $$y_{1}\;=\;-0.1125x^{2}\;+\;0.9748x\;-\;10.9883$$

(2) $$y_{2}=-0.0074x^{2}+0.7612x-8.2143$$

(3) $$y_{3}=-0.0146x^{2}+1.1967x-15.2401$$

(4) $$y_{4}=-0.0057x^{2}+0.5996x-5.6335$$

The fitting functions were used to calculate the welding currents required under different electrode forces to achieve both the minimum acceptable (3√t) and optimal (5√t) nugget diameters, with t being the sheet thickness (3 mm). The calculated values are summarized in Table 2.

Based on the fitted welding current values and the minimum expulsion currents, the operating window for the resistance spot welding of AlSi_7_MnMg non-heat-treatable die-cast aluminum alloy was constructed, as shown in Figure 7b. The region to the left of the black line represents parameter combinations that fail to form the minimum acceptable nugget diameter, while the area to the right of the red line indicates conditions prone to interfacial expulsion, resulting in loss of nugget metal and degradation of joint mechanical properties. Analysis under the condition of 100 ms welding time reveals that an electrode force of 7.5 kN provides the widest overall process window (16.64 kA), allowing for the greatest flexibility in parameter selection. In contrast, an electrode force of 6.5 kN yields the widest interval (7.67 kA) between the current for achieving the optimal nugget diameter and the expulsion limit, which is more conducive to ensuring high-strength joints. In summary, compared to conventional aluminum alloys, the AlSi_7_MnMg alloy exhibits a lower minimum expulsion current and a higher minimum current for nugget formation, resulting in a significantly narrower overall process window and substantially inferior process robustness.

As shown in Table 3, under identical current conditions of 32.5 kA and 40.0 kA, electrode pressure of 5.5 kN generates shear stresses of 6812.41 kN and 6316.33 kN, respectively, with failure modes PF and IF. When electrode pressure is reduced to 6.5 kN, shear stresses increase to 7651.46 kN and 8518.89 kN, corresponding to failure modes MF and BF. This demonstrates that 6.5 kN voltage facilitates higher-quality solder joints. Similarly, at current levels of 40.0 kA and 42.5 kA, 5.5 kN electrode pressure produces shear stresses of 6316.33 kN and 5817.60 kN, respectively, with IF failure mode observed. Notably, significant spatter occurs at 42.5 kA current, whereas 7.5 kN electrode pressure shows no noticeable spatter, generating shear stresses of 7112.71 kN and 6916.43 kN, respectively, with MF and BF failure.

### 3.3. Element Segregation in the Spot Weld Joint

**Figure 8:**
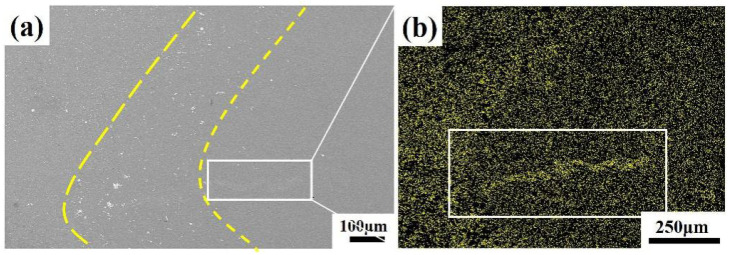
Microstructural characteristics of the resistance spot welding joint under scanning electron microscopy (SEM): (**a**) Second-phase segregation band; (**b**) Si element segregation zone.

To investigate the fracture mechanisms and the root cause of the narrow processing window in AlSi_7_MnMg non-heat-treatable die-cast aluminum alloy spot welds, the joint microstructure was examined using scanning electron microscopy (SEM). Figure 8 displays a typical microstructure observed at location A indicated in Figure 2a. The analysis revealed significant segregation of secondary phases. Additionally, silicon (Si) was found to form a continuous band-like segregation zone.

**Figure 9:**
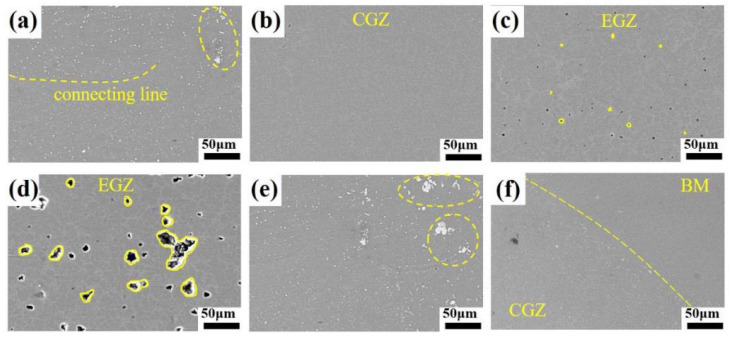
Microstructures of different zones of the resistance spot welding joint under scanning electron microscopy (SEM): (**a**) Connecting line; (**b**) Columnar grain zone; (**c**) Equiaxed fine-grain zone; (**d**) Equiaxed coarse-grain zone; (**e**) Fusion boundary; (**f**) Base metal.

To further analyze the distribution characteristics of secondary phases, magnified examinations were conducted on different regions of the joint, as shown in Figure 9. In the resistance spot weld of the AlSi_7_MnMg non-heat-treatable die-cast aluminum alloy, secondary phases predominantly appear as bright constituents and form a distinct segregation band along the nugget boundary. Observations indicate that the equiaxed grain zone within the nugget contains virtually no secondary phases, while the columnar grain zone exhibits a limited number of fine, particulate secondary phases. In contrast, large secondary phases are almost exclusively concentrated near the nugget boundary. Therefore, the enhanced hardness in the columnar grain zone can be attributed to two primary factors: the notable increase in both the population and size of the hard Al_15_(Fe,Mn)_3_Si_2_ phases in this region, and the prevalent segregation of the inherently hard eutectic silicon phases. Conversely, the comparatively lower content of hard strengthening phases within the equiaxed grain zone also contributes to its reduced hardness.

Studies have indicated that these coarse secondary phases located at grain boundaries can induce significant stress concentration, thereby promoting crack initiation [13,14]. These hard and brittle phases disrupt the continuity of the aluminum matrix, acting as inherent micro-crack sources. During lap-shear testing, stress initially concentrates at the interfaces between these brittle phases and the matrix, leading to the formation of micro-cracks. These cracks readily propagate along this vulnerable segregation band, resulting in low-stress interfacial failure across the bonded area. This failure mode, characterized as typical interfacial fracture, exhibits a strength substantially below the required standard [15,16].

**Figure 10:**
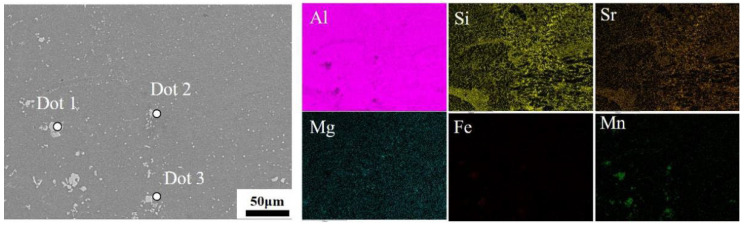
The SE of the spot weld nugget and elemental distribution maps obtained by EDS mapping.

Figure 10 presents the secondary electron (SE) images and the corresponding energy-dispersive X-ray spectroscopy (EDS) elemental maps. The elemental distribution reveals that the spot-welded joint primarily consists of an Al matrix and eutectic Si phases. The eutectic Si phases, characterized by their relatively small size, are predominantly located at the Al grain boundaries. Notably, Si segregation is observed near the nugget boundary. The distribution of Fe and Mn elements, as revealed by EDS mapping, corresponds to the bright secondary phases visible in the SE images. Point analysis results, summarized in Table 3, confirm that these bright secondary phases are Al_15_(Fe,Mn)_3_Si_2_, which is consistent with findings reported in the literature [17,18,19]. In local regions with higher Fe content, the morphology of the Al_15_(Fe,Mn)_3_Si_2_ phase transforms into larger, blocky constituents that aggregate at grain boundaries. During resistance spot welding, liquid metal is expelled toward the edge of the molten pool under the influence of liquid phase expansion forces and electromagnetic repulsion forces. This can lead to the local enrichment of solute elements near the nugget boundary, thereby promoting the formation of additional coarse secondary phases [20,21,22], which severely constrains the processing window.

In the initial stage of spot welding, the presence of coarse eutectic Si and Al_15_(Fe,Mn)_3_Si_2_ phases necessitates that the welding current must first overcome the obstruction posed by these high-resistivity hard phases. Local heating is required to melt or dissolve them before a continuous current path and a unified nugget can be established. This process consumes additional energy, effectively raising the “activation energy” required for forming a sound nugget. Consequently, the minimum current for nugget formation increases, shifting the left boundary of the processing window to the right [23,24,25]. Although this study did not directly measure the dynamic resistance changes during welding, a comparison of the nucleation behaviors of AlSi_7_MnMg alloys with abundant second phases and conventional 6xxx series aluminum alloys with fewer second phases under identical welding parameters revealed that the former generally required 10–15% higher welding current to achieve the same nucleation size. This phenomenon indirectly supports the hypothesis that “high-resistance phases increase the minimum nucleation current”. During the rapid non-equilibrium solidification of spot welding, solute elements such as Si, Fe, and Mn are continuously rejected by the growing columnar grains into the remaining liquid at the periphery of the nugget. This leads to the formation of a solute-enriched region near the nugget boundary, particularly at the interface between the columnar grain zone and the base metal. The alloy composition in this region significantly lowers the actual eutectic temperature. Under resistive heating, this area, still in a solid–liquid coexistence state, is prone to local remelting, forming a continuous liquid film along the nugget periphery with poor mechanical properties. Under electrode pressure, this remelted liquid film is readily torn, enabling expulsion to occur at lower currents compared to conventional aluminum alloys. This phenomenon manifests as a leftward shift in the right boundary of the processing window.

**Figure 11:**
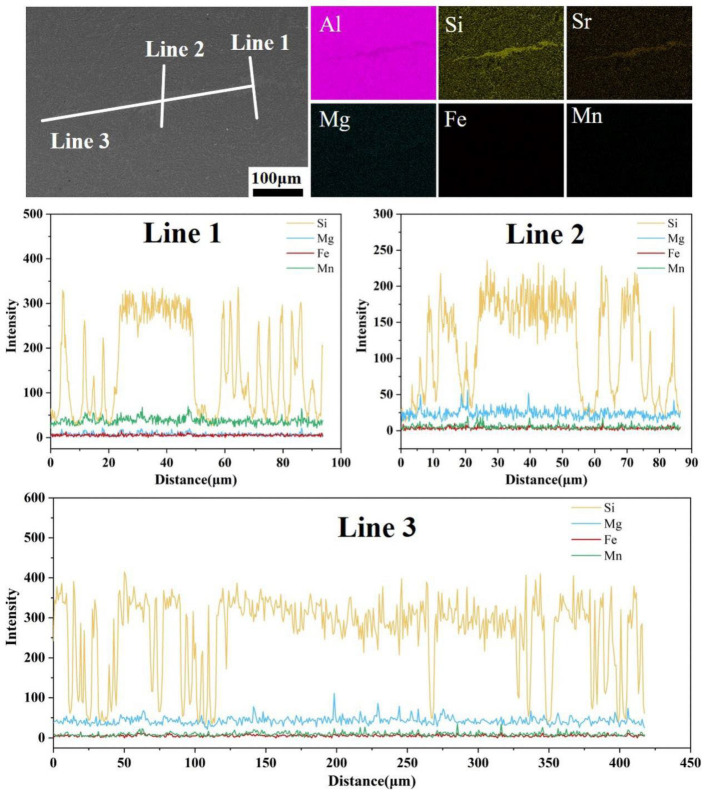
Backscattered electron (BSE) imaging and surface and line scan energy dispersive spectroscopy (EDS) analysis of Si element segregation zones: (Line 1) Vertical direction at the edge of the silicon segregation zone; (Line 2) Vertical direction at the center of the silicon segregation zone; (Line 3) Along the direction of the silicon segregation zone.

To further characterize the elemental composition of silicon segregation zones, surface and line scan energy dispersive spectroscopy (EDS) was performed on the silicon segregation region (Figure 11). The segregation band exhibits a narrow shape on both sides and a wide central section, with a tip width of approximately 26.5 μm and a central width of about 32.0 μm, totaling approximately 417.5 μm in length. The silicon concentration is higher on both sides and lower in the center, with an average tip intensity of 281.6, average central intensity of 172.0, and overall average intensity of 264.9. Comprehensive analysis of morphology and elemental content indicates that the macroscopic silicon segregation phenomenon in the spot weld joint conforms to the characteristics of liquid-phase backfill solidification. Surface and line scan energy dispersive spectroscopy (EDS) analysis revealed the presence of Mg-, Mn-, and Fe-rich strengthening phases within the zone. These phases are closely associated with elemental segregation during the solidification process of the molten pool. The spot scan EDS results (Table 4) confirm that Dot 1–Dot 3 correspond to the Al_15_(Fe,Mn)_3_Si_2_ phase as specified in Table 5, while Dot 4 and Dot 5 represent the eutectic Si phase and α-Al matrix, respectively. Both phases constitute the primary components of the room-temperature AlSi_7_MnMg alloy in the heat-treatment-free die-casting process. Therefore, the Si segregation zone in the spot-welded joints of this alloy primarily consists of the eutectic Si phase and Al_15_(Fe,Mn)_3_Si_2_ phase.

**Table 5:** Elemental composition data (wt.%) of the marked positions in Figure 12 obtained by energy-dispersive spectroscopy (EDS) point scanning.

Points	Al	Si	Sr	Mn	Fe	Mg
Dot 1	78.97	8.72	0.41	10.07	1.72	0.11
Dot 2	76.62	15.58	0.57	4.27	1.14	1.82
Dot 3	76.93	17.49	0.77	2.78	0.67	1.36
Dot 4	79.16	19.21	0.86	0.36	0.06	0.35
Dot 5	96.01	3.31	0.24	0.24	0	0.20

**Figure 13:**
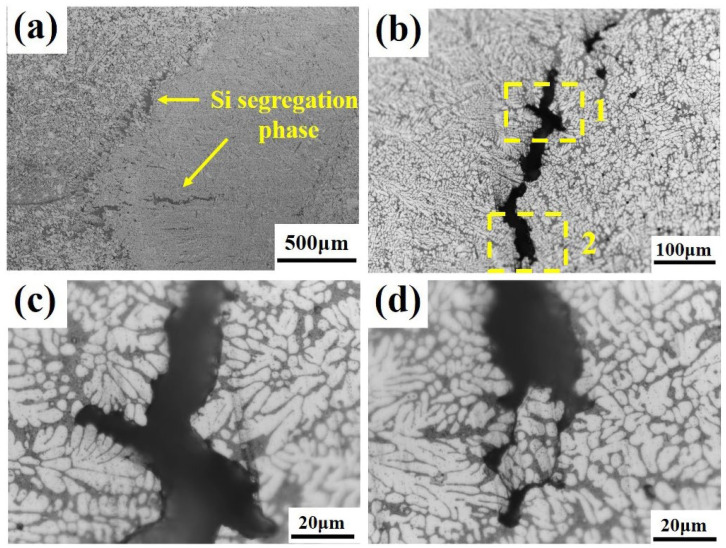
(**a**) Location of Si element segregation; (**b**) Shrinkage porosity defect within the segregation zone; (**c**) Enlarged view of zone 1; (**d**) Enlarged view of zone 2.

**Figure 14:**
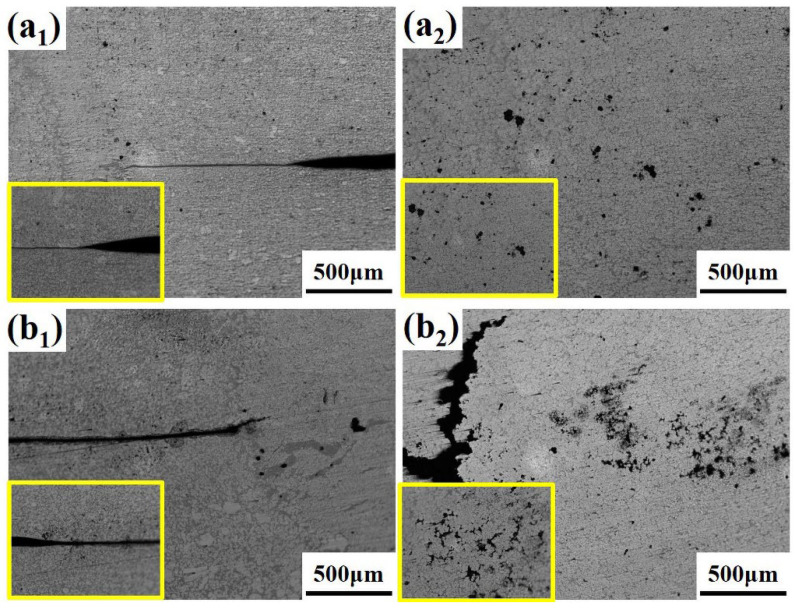
Comparison of fracture paths: (**a_1_**) Bonding line at 50% total strain; (**a_2_**) Central shrinkage pore at 50% total strain; (**b_1_**) Bonding line at 70% total strain; (**b_2_**) Central shrinkage pore at 70% total strain.

Figure 13 reveals the distribution relationship between Si element segregation and shrinkage porosity defects. It can be observed that Si segregation appears as banded or blocky formations, concentrated in the columnar grain zone at the nugget periphery and distributed intermittently along the fusion line. The shrinkage porosity defects are continuously distributed along the trajectory of Si segregation, and their formation mechanism can be attributed to inadequate feeding during the final stages of solidification [26,27,28]. During the rapid non-equilibrium solidification of spot welding, Si, as a solute element, is rejected by the growing columnar grains into the solid–liquid interface front, leading to a continuous increase in Si concentration in the liquid phase. Concurrently, under the influence of liquid phase expansion forces and electromagnetic stirring, the solute-rich melt is pushed toward the outer regions of the nugget, further exacerbating Si segregation in the columnar grain zone. This segregation shifts the local composition closer to the Al-Si eutectic point, significantly depressing the solidification temperature and thereby making the Si-rich zones the last regions to solidify within the nugget. When a complete dendritic network has already formed in the interior of the nugget, the isolated Si-rich liquid pockets located inter-dendritically begin their eutectic reaction. At this stage, the feeding channels have already become severely obstructed. As shown in Figure 14, these feeding channels exhibit a characteristic “narrow at both ends and wider in the middle” morphology. While some degree of liquid back-filling can still occur in the wider central section, forming high-Si eutectic structures, the narrow sections suffer from an imbalance between solidification shrinkage and flow resistance. This results in insufficient feeding capability, ultimately leading to the formation of shrinkage porosity defects distributed along the Si segregation band.

Mechanically, such shrinkage porosity is equivalent to pre-existing micro-voids. Under lap-shear loading, severe stress concentration occurs at the pore tips, making them potent sites for crack initiation [29,30]. Consequently, even when the joint fails via pull-out fracture, the cracks often originate from clusters of shrinkage porosity located inside or at the edge of the nugget. The distribution and severity of the shrinkage porosity directly determine the load-bearing capacity during pull-out fracture: when process optimization results in a larger nugget diameter and confines the porosity primarily to the central region, the resistance to crack initiation increases and the effective load-bearing area is enhanced, leading to a corresponding improvement in lap-shear strength. If the joint strength subsequently surpasses that of the base metal, failure location shifts to the base metal, indicating that the nugget performance has reached a superior state.

Furthermore, even when shrinkage cavities do not initiate cracks, they elongate under loading and interconnect to form new fracture zones. Figure 14 shows the fracture path of the joint under conditions of I = 40 kA, F = 6.5 kN, and t = 100 ms. The results demonstrate that after loading, the bonding line at the outer interface cracks first, while the shrinkage cavity reaction at the melt core center lags behind and shows no significant change initially. As deformation increases, the bonding line gradually extends into the melt core, and the central shrinkage cavity elongates under tensile stress, connecting adjacent cavities to ultimately form a continuous fracture zone. During this fracture process, the tearing failure behavior results from both the extension of the bonding line and the interconnection of aggregated shrinkage cavities.

To further investigate the impact of shrinkage defects on joint load-bearing capacity, this study conducted a qualitative analysis of shrinkage distribution in typical joints. The results indicate that shrinkage primarily concentrates within the silicon segregation zone, with its density showing a negative correlation with tensile shear strength. For instance, in interface fracture specimens subjected to low tensile shear loads, shrinkage exhibits continuous distribution along the melt core boundary. In contrast, in fracture specimens subjected to high tensile shear loads, shrinkage is predominantly confined to the central region of the melt core. Although limited by sample quantity and detection methods, the quantitative relationship between porosity and strength could not be systematically statistically analyzed. Nevertheless, these trends suggest that controlling silicon segregation and shrinkage distribution constitutes one of the key approaches to enhancing joint mechanical properties. Therefore, the distribution and scale of shrinkage cavities directly influence the load level required for fracture initiation: when process optimization increases the melt core diameter and confines shrinkage cavities to the central region, crack initiation resistance increases, the effective load-bearing area expands, and tensile shear strength improves accordingly. If the joint strength surpasses that of the base material, the failure location shifts to the base material, indicating that the melt core has reached a high-performance state.

## 4. Conclusions

This study systematically investigates the microstructural characteristics and mechanical properties of resistance spot-welded joints in non-heat-treatable die-cast AlSi_7_MnMg aluminum alloy, with particular emphasis on the governing role of element segregation and secondary phase behavior in fracture mechanisms and the process window. The main conclusions are as follows:

(1) The weld nugget exhibits a typical dual structure consisting of a columnar grain zone (CGZ) and a central equiaxed grain zone (EGZ). Its formation is governed by the thermal gradient (G), cooling rate (R), and solute concentration (C_0_). The high cooling rate and heterogeneous nucleation at the nugget periphery promote the formation of the CGZ, while dendrite fragmentation, constitutional undercooling, and a lower thermal gradient in the nugget center lead to the development of the EGZ. Variations in the thermal cycle across different regions are the primary cause of the non-uniform grain size and defect distribution within the EGZ.
(2) The “M” -shaped hardness distribution fundamentally reflects the spatial inhomogeneity of second-phase distribution, solidification defects, and grain structure within the melt core. The Al_15_(Fe,Mn)_3_Si_2_ intermetallic compound exhibits significant segregation near the melt core boundary, where its high-density distribution directly contributes to localized hardening. During columnar crystal growth, silicon elements are displaced between dendrites and the liquid-solid interface, forming a fine eutectic Si network that serves as a dispersion strengthening mechanism. Simultaneously, grains in the columnar zone grow along the temperature gradient direction, with higher grain boundary density. Rapid solidification inhibits grain coarsening, further preserving hardness. These factors collectively result in the highest hardness value (101.3 HB) in the columnar zone. In contrast, the equiaxed zone, being the final solidification region, suffers from insufficient shrinkage, leading to multiple porosities. These voids significantly reduce the material’s effective load-bearing area and continuity. Additionally, the absence of Al_15_(Fe,Mn)_3_Si_2_ phase causes severe softening in this region.
(3) Elemental segregation is likely the primary cause of defect formation and performance degradation. Solute elements, including Si, Fe, and Mn, are rejected towards the nugget boundary during rapid solidification, forming a segregation band rich in Al_15_(Fe,Mn)_3_Si_2_ intermetallics and eutectic Si. The severe Si segregation significantly depresses the local solidification temperature, making this region the last to solidify. Inadequate feeding during this final stage results in the formation of shrinkage pores distributed along the segregation band. These segregated brittle phases and shrinkage pores act as inherent crack initiation sites, directly influencing the failure mode of the joint.
(4) At t = 100 ms, the unique element segregation and second-phase elevation raise the minimum current required for forming qualified melt nuclei. In the as-cast microstructure, the coarse eutectic Si-Al_15_(Fe,Mn)_3_Si_2_ phase exhibits high resistivity, requiring additional energy during initial current application to achieve melting or dissolution, thereby increasing the current threshold for effective melt nucleus formation. Meanwhile, the solute segregation during the final solidification stage creates a low-melting-point zone, enabling spatter to occur at lower currents and thus lowering the upper limit of the process window. Compared to conventional aluminum alloys, this material demonstrates significantly narrowed spot welding process windows and poor process stability. The key to optimizing the process lies in precise thermal input control to suppress detrimental segregation, ultimately yielding high-performance joints with properties approaching or even surpassing those of the base material.

## Figures and Tables

**Figure 12:**
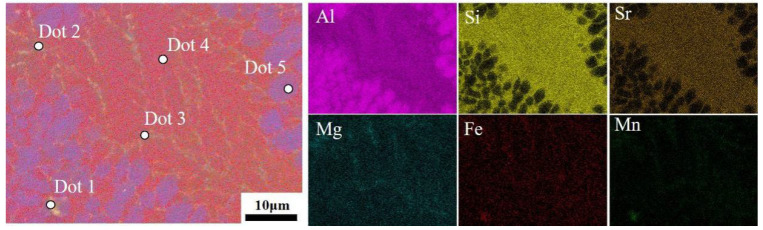
Energy-dispersive spectroscopy (EDS) analysis of the second phase within the Si segregation band.

**Table 1:** Chemical composition of AlSi_7_MnMg aluminum alloy (wt.%).

Ingredient	Si	Fe	Mn	Mg	Ti	Sr	Al
Content(wt.%)	7.12	0.13	0.61	0.21	0.05	0.008	Remaining

**Table 2:** Fitted welding current values corresponding to different electrode pressures.

*y* (mm)	*x*_1_ (kA)	*x*_2_ (kA)	*x*_3_ (kA)	*x*_4_ (kA)
6.06 (3√t)	24.3782	24.6680	26.1274	25.8589
8.66 (5√t)	32.1033	32.3279	34.4558	36.5105

**Table 3:** Shear Stress and Failure Modes under Different Welding Parameters.

Electrode Force (kN)	Welding Current (kA)	Nugget Diameter (mm)	Lap-Shear Load (N)	Failure Mode
5.5	32.5	8.76	6812.41	PF
5.5	40.0	9.53	6316.33	IF
5.5	42.5	10.47	5817.60	IF
6.5	32.5	8.41	7651.46	MF
6.5	35.0	8.71	7866.24	MF
6.5	40.0	11.08	8518.89	BF
7.5	40.0	8.44	7112.71	MF
7.5	42.5	10.06	6916.43	MF

**Table 4:** Elemental composition data (wt.%) of the marked positions in Figure 8 obtained by energy-dispersive spectroscopy (EDS) point scanning.

Points	Al	Si	Sr	Mn	Fe	Mg
Dot 1	59.00	11.61	0.57	24.39	4.38	0.05
Dot 2	70.43	12.95	0.17	14.1	2.24	0.12
Dot 3	69.83	13.19	0.17	14.42	2.26	0.13

## Data Availability

The original contributions presented in this study are included in the article. Further inquiries can be directed to the corresponding author.
